# The School Suicide Policy Evaluation Tool (SSPET): A proof‐of‐concept for assessing school suicide prevention policies

**DOI:** 10.1002/puh2.178

**Published:** 2024-05-02

**Authors:** Douglas Wirthlin, Zeke Richards, Cody S. Crandall, Shad P. Mortensen, Jackson R. Richards, Amanda V. Bakian

**Affiliations:** ^1^ Rocky Vista University School of Osteopathic Medicine Parker Colorado USA; ^2^ The Department of Psychiatry The University of Iowa Carver College of Medicine Iowa City Iowa USA; ^3^ Department of Psychiatry The Huntsman Mental Health Institute, University of Utah Salt Lake City Utah USA

**Keywords:** evaluation, health policy, school, suicide prevention

## Abstract

**Background:**

As adolescent suicide rates have increased, there has been a nationwide increase in legislation requiring schools to create suicide prevention policies. In Utah, school districts must implement a youth suicide program for grades 7–12. Currently, there have been no systematic analyses of Utah school district suicide prevention policies. Thus, we developed a new evaluation instrument, the School Suicide Policy Evaluation Tool (SSPET).

**Methods:**

The SSPET was derived from the “Model School District Policy” and evaluates criteria on prevention, intervention, re‐entry, and postvention. Four raters used the SSPET in a systematic analysis of suicide prevention policies for all school districts in the state of Utah.

**Results:**

Analysis revealed a right‐skewed distribution of policy scores, and a median total score was 2.25/36. The mean percentage of inclusion of the four subsections in policies was less than 20% for most criteria. Median scores were totaled for the four subsections as follows: Prevention score was 1/9, intervention score was 1/14, re‐entry score was 0/4, and postvention score was 0/9.

**Conclusion:**

This proof‐of‐concept study demonstrates the utility of the SSPET in identifying shortcomings of school district policies on suicide while generating specific recommendations for improvement. With further validity testing, we expect this tool to be useful to school district administrators, education policymakers, and those studying suicide interventions and outcomes in school‐aged children and adolescents.

## Graphical abstract

**Study aim**: The School Suicide Policy Evaluation Tool (SSPET) was created to analyze school district suicide policies based on the national standard “Model School District Policy.” The tool was statistically verified and then used to analyze the state of Utah's school district policies.

**Conclusion**: Overall performance for school districts in Utah was poor, with over three quarters of districts receiving a total score of less than 4 out of a possible 36. This proof‐of‐concept study demonstrates the utility of the SSPET in identifying shortcomings of school district policies on suicide while generating specific recommendations for improvement.

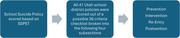

## INTRODUCTION

Suicide rates of individuals aged 10–24 years have increased by 57.4% from 2008 to 2017 [1]. Among American adolescents age groups 10–14 years, suicide is the second leading cause of death, and among age groups 15–24 years, suicide is the third leading cause of death [2]. Recent work using a nationally representative sample of nearly 200,000 high school students found that one in five endorsed suicidal ideation and 14.7% had a suicide plan [3]. This issue is especially pertinent in Utah, where the incidence of suicide is 16.6/100,000 in people aged 10–24 years, well above the national average of 10.3/100,000 [1]. There is a substantial amount of research underway to understand the increasing incidence of suicidal ideation and behaviors in the youth, adolescent, and young adult populations and its connection to school‐based behavioral health interventions [3, 4, 5, 6, 7, 8, 9]. However, the factors contributing to increased suicide rates and their relationship with best practices in the school setting are incompletely understood.

The school setting is an ideal location to implement suicide prevention programs because it reaches most children of school age, and many risk factors for suicide are related to the school setting. However, the nuances of risk factors vary based on ethnicity; some of the risk factors include, bullying, school connectedness, academics, program effects, and in‐school behavior [10]. School‐based suicide prevention programs are widely used and a popular framework for addressing adolescent suicidality [11].

As awareness around youth and adolescent suicide increases, elected representatives nationwide are passing legislation aimed at reducing suicidal thoughts and behaviors in these age groups [12, 13, 14]. One area of emphasis is improving school‐based suicide prevention policies. Several states across the nation now legally require school districts to have suicide prevention policies in place [15]. In Utah, school districts have been required to allocate funds for suicide prevention programs since 2018. However, there is no specific requirement regarding the codification of these programs into district policy [16]. An important point of distinction for the current study is the difference between school‐level suicide prevention programs and school district suicide policies. The former are measures carried out by a school's faculty, and the latter are guidelines determined by school districts by which school‐based suicide prevention programs are created for each school.

Recent work has concluded that school‐based suicide prevention programs lead to a modest but significant reduction in suicidal ideation and behaviors in youth and adolescents [17, 18]. These studies include meta‐analyses that assessed the effects of various suicide prevention programs, such as Signs of Suicide, HeadStrong, and Question Persuade Refer. However, there has not been nationwide adoption of any particular program [19]. A critical question is whether more generalized principles and practices as outlined in school‐based suicide prevention policies might also be effective in reducing suicidal ideation and behaviors in this population. This is especially pressing given that national suicide rates among adolescents continue to rise [20, 21, 22]. Before policy implementation and outcomes can be assessed, the content of the policies themselves must be scrutinized [23]. To systematically conduct such an assessment, a robust evaluation tool is needed. Ideally, it would be easy to use, applicable throughout the country, and examine the various facets of school policy of the prevention of and response to student suicide. To our knowledge, this type of tool for assessing school‐based suicide prevention policies has not been previously published.

There is, however, precedent for such a policy evaluation tool in school health and wellness. The CDC's Whole School, Whole Community, Whole Child (WSCC) model was used to create an assessment tool called the Wellness School Assessment Tool (WellSAT). This tool aids school districts in evaluating the alignment of the WSCC model and their district policies regarding nutrition and physical education [24]. We took a similar approach and developed a tool for the evaluation of school‐based policies on suicide prevention based on consensus recommendations found in the *Model School District Policy on Suicide Prevention* (MSDP). This document was created in collaboration with the American Foundation for Suicide Prevention, the American School Counselor Association, the National Association of School Psychologists, and The Trevor Project [25]. The MSDP “outlines model policies and best practices for school districts to follow” [25] regarding suicide prevention, intervention, re‐entry, and postvention. This model policy was assembled by a host of mental health professionals, including psychiatrists, school psychologists, a Ph.D. in psychiatric epidemiology, as well as various experts in government policy and advocacy. The MSDP was created by reviewing policies already in place across the country in K‐12 schools and identifying the strengths of each to create a national model. The scope of the MSDP covers suicide intervention at school and school‐related functions for students, parents, and school faculty. At the core of the policy, its stated purpose is to “protect the health and well‐being of all students by having procedures in place to prevent, assess the risk of, intervene in, and respond to suicide” [25]. Many states’ Boards of Education, including Utah's, provide the MSDP as a resource for districts to develop suicide prevention policies. Given its comprehensive yet modular design, the MSDP is an ideal basis for a tool to evaluate the content of school district suicide prevention policies. Indeed, the MSDP is regarded as the primary resource for guidelines on making suicide prevention school district policies [26]. An assessment tool based on the guidelines of the MSDP could highlight policy deficiencies and strengths as well as monitor trends across many district policies.

The objectives of the study are (1) to create an instrument, the School Suicide Policy Evaluation Tool (SSPET), for the quantitative evaluation of school district policies on suicide prevention, intervention, re‐entry, and postvention, and (2) to deploy this tool in an analysis of suicide prevention intervention, re‐entry, and postvention policies for all school districts in the state of Utah.

## METHODS

### SSPET development

The MSDP was used to identify 36 specific, nonredundant criteria to assess school district suicide prevention policies. Following the MSDP, these criteria were divided into the subsections of prevention (9 criteria), intervention (14 criteria), re‐entry (4 criteria), and postvention (9 criteria; see Table 1). We intended to create a proof‐of‐concept measurement, and so a secondary editor of the MSDP reviewed the SSPET and confirmed the face validity of the tool, affirming that it evaluates best practices described in the model policy. To generate scores, a policy is evaluated for the presence (one point) or absence (zero points) of each criterion. The total policy score is the sum of individual scores from each subsection for a maximum possible score of 36.

**TABLE 1:** The School Suicide Policy Evaluation Tool (SSPET).

Prevention (Pr)			
The policyƈ		Yes	No
Pr‐1	Indicates that the district will appoint a district suicide prevention coordinator		
Pr‐2	Indicates who will designate a school suicide prevention coordinator		
Pr‐3	Mandates that all staff members will report students they believe to be at risk		
Pr‐4	Indicates how often professional development courses on suicide prevention are required for all staff		
Pr‐5	Requires professional development for all staff regarding groups of students at elevated risk for suicide (substance use, mental health issues, history of self‐harm, out‐of‐home settings, experiencing homelessness, American Indian/Alaska native students, LGBTQ students, and medical conditions/disabilities)		
Pr‐6	Indicates any additional professional development in risk assessment and crisis intervention for school mental health professionals and nurses		
Pr‐7	Requires developmentally appropriate, student‐centered education materials addressing the warning signs of mental health conditions and suicide, as well as help‐seeking strategies for oneself and peers in the curriculum of all K‐12 health classes		
Pr‐8	Explains the distribution and publication of the policy		
Pr‐9	Indicates whether school personnel will be accountable for knowing the policy		
**Prevention (Pr) Score**			**/9**

Intervention (I)			
The policyƈ		Yes	No
I‐1	Indicates a timeframe in which a social mental health professional must see a student who has been identified as at risk for suicide (i.e., verbalizes thoughts about suicide, presents overt risk factors such as agitation or intoxication, an act of self‐harm occurs, or expresses or otherwise shows signs of suicidal ideation)		
I‐2	Includes a protocol for written threats and expressions about suicide and death in school assignments		
I‐3	Indicates who will address the situation if there is no mental health professional available		
	For at‐risk youth, the policyƈ	Yes	No
I‐4	Addresses the supervision of the student while the assessment is being completed		
I‐5	States that the principal and school suicide prevention coordinator be made aware as soon as possible		
I‐6	Includes a protocol for parental notification		
I‐7	Includes a protocol for an urgent referral to an outside mental health provider		
I‐8	Includes a protocol for when parental abuse or neglect is suspected		
I‐9	Includes a protocol for obtaining written permission from student's parents or guardians to discuss the student's health with outside care providers		
I‐10	Addresses situations when the school personnel need to engage law enforcement, specifically officers with training in crisis de‐escalation and mental illness		
I‐11	Includes a timeline for when school employees will notify the parents or guardians		
I‐12	Includes guidelines for what recommendations school staff may offer to parents or guardians		
I‐13	Includes guidelines for “safety at home” information to be shared with the parents or guardians		
I‐14	Includes guidelines for lethal means counseling for parents or guardians		
**Intervention (I) Score**		/14	

Re‐entry (R)			
The policyƈ		Yes	No
R‐1	Indicates that a school employee will meet with the parents or guardians of a student who is returning to school after a suicide attempt		
R‐2	Includes guidelines for re‐entry of the student after suicide attempt		
R‐3	States that a designated staff will periodically check in with the student and their parents or guardians at an agreed‐upon interval		
R‐4	Instructs that teachers be informed that the student is returning from a medically‐related absence		
**Re‐entry (R) Score**		**/4**	

Postvention (Po)			
The policyƈ		Yes	No
Po‐1	Includes a protocol for in‐school suicide attempts		
Po‐2	Includes a protocol if a staff member becomes aware of an out‐of‐school suicide attempt		
Po‐3	Includes guidelines for a postvention plan in the event of a suicide		
Po‐4	Includes guidelines for confirming the suicide and communicating with the parents		
Po‐5	Includes having a crisis response team in schools		
Po‐6	Includes guidelines regarding how and when information on the suicide will be shared with faculty and students		
Po‐7	Includes strategies to avoid suicide contagion		
Po‐8	Includes guidelines for initiating support services for faculty and students		
Po‐9	Includes guidelines for the development of memorial plans for the deceased student		
**Postvention (Po) score**		**/9**	
**Total (Pr + I + R + Po) score**		**/36**	

*Note*: The evaluation instrument is divided into four subsections—prevention (Pr), intervention (I), re‐entry (R), and postvention (Po). Each item is worth one point. The sum of the four subsections is the total score (the maximum possible score is 36).

### Policy collection

A school district in the United States is a geographically defined administrative region responsible for overseeing and managing public education in the schools within its boundaries. The district's primary role is to provide educational services to students residing in its designated area, ensuring compliance with state and federal education regulations. Utah state law requires that all school districts make school board‐approved policies publicly available on their school district website [27]. School board–approved district policies regarding suicide prevention were obtained from district websites for all school districts in the state of Utah. Utah school district policies encompass a range of categories, including local district governance, business and support services, personnel, instruction, students, and community. The information regarding suicide prevention was found in the student section of all district policies. All policies were approved and voted in by the school district board of each school district. School district administrators were contacted to confirm the collection of all school district policies related to suicide prevention, intervention, postvention, and re‐entry. A list of URLs of district policies is provided in Table S1.

### Policy evaluation

Four graduate‐level students with minimal training in behavioral health used the SSPET to score the policies of all 41 school districts in the state of Utah independently. Three of the four raters were not involved in the creation of the SSPET and had no exposure to the MSDP before its use. Each of the four raters independently submitted their scores for each criterion for subsequent analysis.

### Statistical analysis

The program R Studio (version 1.4.1717) was used to evaluate inter‐rater reliability using intraclass correlation analysis with a two‐way random‐effects model (irr package). This allowed for an assessment of the agreement among raters and to determine how much of the total variance could be attributed to a single component. Subsection and total scores for each district were calculated as the mean of the scores from the four raters. Total score distribution for district policies was assessed by a histogram generated in R. For each subsection and total scores, median values with interquartile ranges were calculated and plotted using Prism (version 9). For individual criterion analyses, the percentage of policies that included a particular criterion was calculated for each rater. The mean and standard error for each criterion were calculated and plotted using Prism.

## RESULTS

### Inter‐rater reliability

The complete SSPET is displayed in Table 1. Inter‐rater reliability was 98.8% (95% CI 98.1%–99.3%).

### Distribution of total scores

**FIGURE 1:**
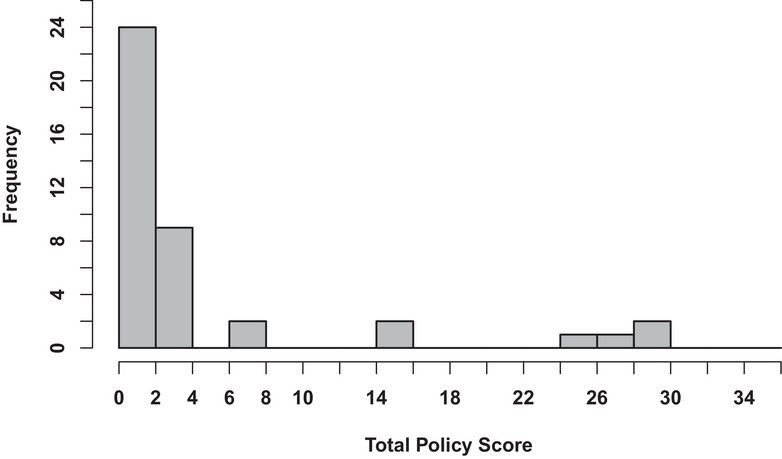
Total scores for suicide policies in Utah school districts. *N* = 41 districts.

SSPET total scores were plotted in a histogram as shown in Figure 1. A right‐skewed distribution was observed, with over 80% of districts receiving a total score of 4 or less out of 36. Four districts scored substantially higher, with total scores between 24 and 30. A higher score denotes a more complete policy. The median total score was 2.25 (first to third quartile ranges: 1.25–3.375).

### Distribution of subsection scores

**FIGURE 2:**
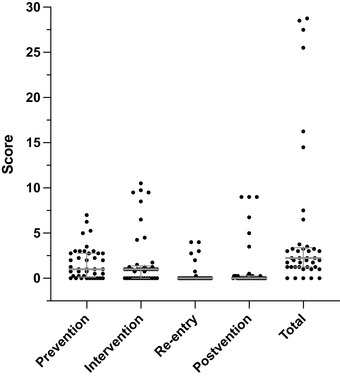
Scatter plot of subsection and total scores for evaluation of suicide policies of Utah school districts. The median scores with interquartile ranges are displayed in gray. *N* = 41 districts.

To further characterize district policy quality, we analyzed scores for the four subsections of prevention, intervention, re‐entry, and postvention (Figure 2). Plotting revealed a similar trend to what was observed for total scores. Most districts received low scores, but there were several outliers with much higher subsection scores. Scores for re‐entry and postvention were low across districts, with a median score of zero in each of these subsections. Median scores out of the total possible (first to third quartile ranges) were as follows for the four subsections: prevention 1.0/9.0 (0.125–2.75), intervention 1.0/14.0 (0–1.375), re‐entry 0.0/4.0 (0.0–0.0), and postvention 0.0/9.0 (0.0–0.25).

### Individual criterion analysis

**FIGURE 3:**
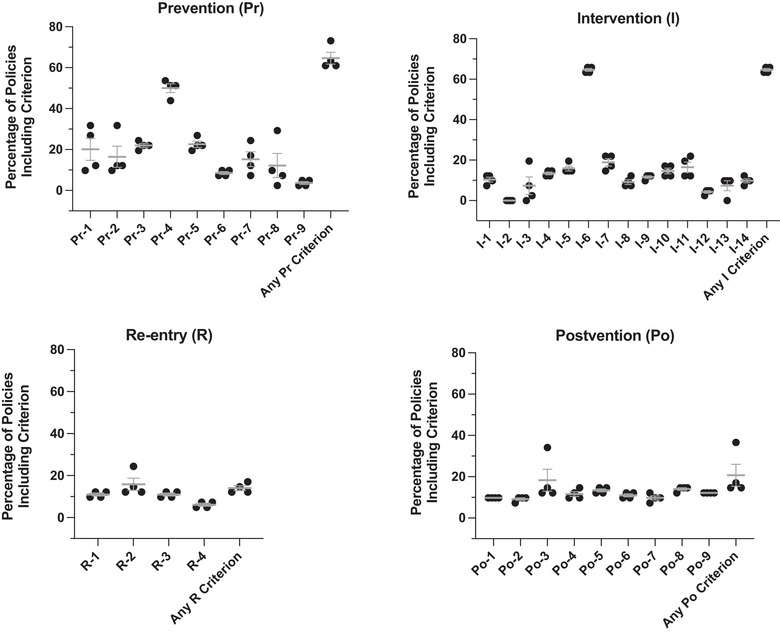
Individual criteria analysis. Scatter plots demonstrating the percentage of Utah school district policies (*N* = 41) that included individual criteria for each of the four subsections. The mean and SEM are displayed in gray. *n* = 4 raters.

To obtain an even more granular assessment of policy content, the percentage of policies, including each criterion, was calculated (Figure 3). The mean percentage of inclusion in policies was less than 20% for most criteria. Percentages were even lower for re‐entry and postvention. The plot revealed two outlier criteria, one in the prevention and one in the intervention subsections. Most district policies indicate how often professional development courses on suicide prevention are required (Pr‐4) and include a protocol for parental notification in the event of a suicide (I‐6).

## DISCUSSION

### Findings from analysis of policies in the state of Utah

Here we present the SSPET, a proof‐of‐concept instrument for comprehensively evaluating school‐based policy content about suicide prevention, intervention, re‐entry, and postvention. It has high inter‐rater reliability (98.8%), even among raters with no prior experience in suicide prevention research. The use of the SSPET in an analysis of the policies of all school districts in the state of Utah resulted in several important findings. Overall performance was poor, with over three‐quarters of districts receiving a total score of less than 4 out of a possible 36. There were, however, several outlier districts that had much more comprehensive policies and, therefore, higher scores. The use of the SSPET also highlighted re‐entry and postvention as areas of especially poor performance. Lastly, the following two criteria, “the policy indicates how often professional development courses on suicide prevention are required for all staff” and “the policy includes a protocol for parental notification,” were more consistently included in district policies.

Several factors could be contributing to overall low scores, including state laws with potentially vague or unenforced standards, growing opposition toward social and emotional learning modalities in schools, or insufficient personnel knowledge in policy creation [28, 29, 30]. The low scores may be at least partially explained by the fact that Utah state law requires school districts to have suicide prevention programs but does not stipulate that these be reflected in school board‐approved policies [16]. Four districts scored substantially higher than the rest, though the reason for this is not readily apparent. It is possible that the leaders of these districts were aware of the MSDP; communication with them might shed light on their higher performance and yield valuable insights for other districts.

One advantage of the SSPET is its ability to highlight specific areas of policy deficiency and strength. In our analysis of Utah school district policies, subsection scores for re‐entry and postvention were particularly low. This is troubling because the period following a psychiatric hospitalization is critical, as the student is at heightened risk for subsequent hospitalizations [31]. Specifying re‐entry procedures is necessary for coordinating the efforts of all teachers and faculty involved at the time of re‐entry [32, 33, 34]. Likewise, specific protocols for postvention protect the mental health of all students and may prevent further suicides, including suicide contagion [35, 36, 37]. Districts may utilize the SSPET and principles of the MSDP to strengthen policies regarding these critical processes.

In addition to subsection scores, the SSPET provides useful information on individual criteria. For instance, as shown in Figure 3, none of the school districts mention procedures for responding to written threats of suicide (criterion I‐2). This could indicate poor preparedness to respond to such a scenario. On the other hand, the analysis identified two criteria with unusually high inclusion rates. Most policies included guidelines regarding professional development training (Pr‐4) and parental notification (I‐6). One possible explanation is that these principles are discussed in a template for bullying policy presented by the Utah State Board of Education [38]. This may suggest that resources provided by state agencies influence the comprehensiveness of localized district policies and highlight the importance of coordination between state and district‐level entities [30].

## LIMITATIONS, IMPLICATIONS, AND FUTURE DIRECTIONS

We recognize some important limitations in our development and use of the SSPET. The inter‐rater reliability of the tool was determined from a small number of users on a relatively modest number of total districts in a single state. Further evaluation of the tool by more diverse users across a sample set with broader geographic variability is needed. Further analysis by experts in suicide, school policy, or contributors to the model policy itself would help determine the validity of the tool and add credibility to the efficacy of the MSDP. Factor analysis may also be beneficial in identifying potentially interdependent criteria and resulting in the simplification of the SSPET.

Additionally, the SSPET is only designed to assess school district policies, not school‐level programs, on suicide prevention. This is an important distinction, both generally and in our statewide analysis. Much of the evidence for suicide prevention in schools focuses on specific interventions, including universal, selected, and targeted programs [22]. Further work will be necessary to determine whether there is a correlation between the comprehensiveness of school‐based suicide prevention policies and the adoption of evidence‐based prevention programs.

Considering these limitations and the alarming trends in adolescent suicide, we see the SSPET being utilized by policymakers and researchers alike once more rigorous validity and reliability testing is complete. The tool may assist school district leadership in evaluating and revising their policies to reflect best practices. Effective suicide prevention requires an immense amount of coordination that involves prevention and intervention strategies throughout the years students are in the education system [22]. School districts are in a unique position to coordinate suicide prevention efforts across schools. This makes comprehensive and effective school district policies on suicide vital to preventing suicide. Additionally, cultural considerations play a vital role in suicide prevention [10]. The SSPET does not provide rigid guidelines that ignore these factors; instead, it provides a framework for suicide prevention programs to be implemented in culturally competent ways. Prevention question 5 (PR‐5) discusses professional training for staff regarding students at elevated risk for suicide, and intervention questions 4–14 (I‐4–14) discuss guidelines specifically for at‐risk youth. These cultural considerations, which may otherwise be overlooked, are necessary for developing a comprehensive and effective policy.

From a public health perspective, the quantitative nature of the SSPET allows for a nuanced analysis of an individual district's policy and the discovery of policy trends across different districts. One challenge in studying school‐based suicide prevention efforts is the heterogeneity of programs and organizations involved; the SSPET provides a uniform metric for assessing policy content addressing this complex issue. Further work may help elucidate the potential relationship between policy content as assessed via the SSPET and policy implementation and, ultimately, outcomes of suicidal thoughts and behaviors at the school and district levels.

## CONCLUSION

This study reports the development of the SSPET, a proof‐of‐concept instrument based on the Model School District Policy for the quantitative assessment of school‐based suicide prevention policies. In an analysis of districts in the state of Utah, the SSPET identified specific policy strengths and weaknesses as well as statewide trends. Once further testing is completed on the reliability and validity of SSPET in a larger sample size, it is therefore likely to be of great value to schools across the country as they seek to be more comprehensive in their suicide prevention efforts.

## AUTHOR CONTRIBUTIONS

The content of this publication is solely the responsibility of the authors and does not necessarily represent the official view of funding agencies or organizations involved in the development of the Model School District Policy. All authors reviewed the final manuscript. Amanda Bakian is supported by the Wheeler Foundation.

## ACKOWLEDGMENTS

The authors would like to thank Keygan Miller, M.Ed., for reviewing the policy analysis tool and offering suggestions to improve the phrasing of some questions. The authors would also like to acknowledge Mary Wilde, M.D., for input on the manuscript and Mark Payton, Ph.D., for assistance with statistical analyses.

## CONFLICT OF INTEREST STATEMENT

The authors have no financial or other conflicts of interest to disclose.

## ETHICS STATEMENT

We used publicly available school district policies that are required to be accessible by law for data analysis; thus, neither human nor animal subjects were involved in the study, and ethical approval was not necessary.

## Supporting information

Supporting Information

## Data Availability

We used publicly available school district policies that are required to be accessible by law for data analysis; thus, neither human nor animal subjects were involved in the study, and ethical approval was not necessary.

## References

[1] Centers for Disease Control and Prevention . National Vital Statistics Reports. Centers for Disease Control and Prevention; 2020. Accessed March 13, 2023. https://www.cdc.gov/nchs/products/nvsr.htm

[2] Centers for Disease Control and Prevention . WISQARS Leading Causes of Death Visualization Tool. Centers for Disease Control and Prevention; 2023. Accessed March 13, 2023. https://wisqars.cdc.gov/data/lcd/home

[3] Lindsey MA , Sheftall AH , Xiao Y , Joe S . Trends of suicidal behaviors among high school students in the United States: 1991–2017. Pediatrics. 2019;144(5):e20191187. doi:10.1542/peds.2019-1187 31611338 PMC7299440

[4] Bridge JA , Horowitz LM , Fontanella CA , et al. Age‐related racial disparity in suicide rates among US youths from 2001 through 2015. JAMA Pediatr. 2018;172(7):697‐699. doi:10.1001/jamapediatrics.2018.0399 29799931 PMC6137506

[5] De Silva DA , Diduk‐Smith RM . Comparison of suicides among younger and older adolescents in Virginia, 2008–2017. Arch Suicide Res. 2022;26(4):1958‐1965. doi:10.1080/13811118.2021.1965929 34425060

[6] Kimball D , Bonds S , Brady JP , Blashill AJ . Suicidality, sexual orientation, and race/ethnicity: results from a U.S. representative adolescent sample. Arch Suicide Res. 2022;26(4):1950‐1957. doi:10.1080/13811118.2021.1965928 34459367

[7] Price JH , Khubchandani J . The changing characteristics of African‐American adolescent suicides, 2001–2017. J Community Health. 2019;44(4):756‐763. doi:10.1007/s10900-019-00678-x 31102116

[8] Shain BN . Increases in rates of suicide and suicide attempts among black adolescents. Pediatrics. 2019;144(5):e20191912. doi:10.1542/peds.2019-1912 31611337

[9] Xiao Y , Cerel J , Mann JJ . Temporal trends in suicidal ideation and attempts among US adolescents by sex and race/ethnicity, 1991–2019. JAMA Netw Open. 2021;4(6):e2113513. doi:10.1001/jamanetworkopen.2021.13513 34125218 PMC8204211

[10] Marraccini ME , Griffin D , O'Neill JC , et al. School risk and protective factors of suicide: a cultural model of suicide risk and protective factors in schools. School Psych Rev. 2022;51(3):266‐289. doi:10.1080/2372966X.2020.1871305 35935591 PMC9354860

[11] Fabiano GA , Evans SW . Introduction to the special issue of school mental health on best practices in effective multi‐tiered intervention frameworks. School Mental Health. 2018;11(1):1‐3. doi:10.1007/s12310-018-9283-2

[12] Congress.Gov . Suicide Training and Awareness Nationally Delivered for Universal Prevention Act of 2021 or the STANDUP Act of 2021. 117Th Congress. S.1543 ‐ 117th Congress (2020–2021). Congress.Gov; 2021. Accessed March 13, 2023. https://www.congress.gov/bill/117th‐congress/senate‐bill/1543

[13] Congress.Gov . National Suicide Hotline Designation Act of 2020. 116th Congress. S.2661 ‐ 116th Congress (2019–2020). Congress.Gov; 2023. Accessed March 13, 2023. https://www.congress.gov/bill/116th‐congress/senate‐bill/2661

[14] Cerulli C , Winterfeld A , Younger M , Krueger J . Public health law strategies for suicide prevention using the socioecological model. J Law Med Ethics. 2019;47:31‐35. doi:10.1177/1073110519857312 31298117

[15] National Association of State Boards of Education . State Policy Database—Suicide Prevention Policy. National Association of State Boards of Education; 2019. Accessed 2023. https://statepolicies.nasbe.org/

[16] Utah Legislature . Substances and Mental Health Act. Utah Code Section 62A‐15‐103. Utah Legislature; 2022. Accessed March 13, 2023. https://le.utah.gov/xcode/Title62a/Chapter15/62a‐15‐S103.html

[17] Gijzen MWM , Rasing SPA , Creemers DHM , Engels R , Smit F . Effectiveness of school‐based preventive programs in suicidal thoughts and behaviors: a meta‐analysis. J Affect Disord. 2022;298(Part A):408‐420. doi:10.1016/j.jad.2021.10.062 34728296

[18] Walsh EH , McMahon J , Herring MP . Research review: the effect of school‐based suicide prevention on suicidal ideation and suicide attempts and the role of intervention and contextual factors among adolescents: a meta‐analysis and meta‐regression. J Child Psychol Psychiatry. 2022;63(8):836‐845. doi:10.1111/jcpp.13598 35289410 PMC9544521

[19] Breux P , Boccio DE . Improving schools' readiness for involvement in suicide prevention: an evaluation of the creating suicide safety in schools (CSSS) workshop. Int J Environ Res Public Health. 2019;16(12):2165. doi:10.3390/ijerph16122165 31248082 PMC6617090

[20] Kutcher S , Wei Y , Behzadi P . School‐ and community‐based youth suicide prevention interventions: hot idea, hot air, or sham? Can J Psychiatry. 2017;62(6):381‐387. doi:10.1177/0706743716659245 27407073 PMC5455865

[21] Mishara BL , Stijelja S . Trends in US suicide deaths, 1999 to 2017, in the context of suicide prevention legislation. JAMA Pediatr. 2020;174(5):499‐500. doi:10.1001/jamapediatrics.2019.6066 32065613 PMC7042911

[22] Singer JB , Erbacher TA , Rosen P . School‐based suicide prevention: a framework for evidence‐based practice. School Mental Health. 2019;11:54‐71. doi:10.1007/s12310-018-9245-8

[23] Funk M , Freeman M . Framework and methodology for evaluating mental health policy and plans. Int J Health Plann Manage. 2011;26(2):134‐157. doi:10.1002/hpm.1049 20680967

[24] Koriakin TA , McKee SL , Schwartz MB , Chafouleas SM . Development of a comprehensive tool for school health policy evaluation: the WellSAT WSCC. J Sch Health. 2020;90(12):923‐939. doi:10.1111/josh.12956 33184889 PMC10286646

[25] American Foundation for Suicide Prevention . Model School District Policy on Suicide Prevention. American Foundation for Suicide Prevention; 2022. Accessed March 12, 2023. https://afsp.org/model‐school‐policy‐on‐suicide‐prevention/

[26] Ackerman JP , Horowitz LM . Youth suicide prevention and intervention: best practices and policy implications. Springer; 2022.

[27] Utah Legislature . Local School Board Powers and Miscellaneous Duties. Utah Code Section 53G‐4‐402. Utah Legislature; 2021. Accessed March 12, 2023. https://le.utah.gov/xcode/Title53G/Chapter4/53G‐4‐S402.html

[28] Chriqui JF , Leider J , Temkin D , Piekarz‐Porter E , Schermbeck RM , Stuart‐Cassel V . State laws matter when it comes to district policymaking relative to the whole school, whole community, whole child framework. J Sch Health. 2020;90(12):907‐917. doi:10.1111/josh.12959 33184878 PMC7702124

[29] Haymovitz E , Houseal‐Allport P , Lee RS , Svistova J . Exploring the perceived benefits and limitations of a school‐based social—emotional learning program: a concept map evaluation. Children & Schools. 2018;40(1):45‐54. doi:10.1093/cs/cdx029

[30] Smith‐Millman MK , Flaspohler PD . School‐based suicide prevention laws in action: a nationwide investigation of principals’ knowledge of and adherence to state school‐based suicide prevention laws. School Mental Health. 2019;11:321‐334. doi:10.1007/s12310-018-9287-y

[31] Chung DT , Ryan CJ , Hadzi‐Pavlovic D , Singh SP , Stanton C , Large MM . Suicide rates after discharge from psychiatric facilities: a systematic review and meta‐analysis. JAMA Psychiatry. 2017;74(7):694‐702. doi:10.1001/jamapsychiatry.2017.1044 28564699 PMC5710249

[32] Marraccini ME , Drapeau CW , Stein R , et al. Characterizing children hospitalized for suicide‐related thoughts and behaviors. Child Adolesc Ment Health. 2021;26(4):331‐338. doi:10.1111/camh.12454 33779031 PMC8476654

[33] Marraccini ME , Pittleman C . Returning to school following hospitalization for suicide‐related behaviors: recognizing student voices for improving practice. School Psych Rev. 2022;51(3):370‐385. doi:10.1080/2372966x.2020.1862628 36034937 PMC9400799

[34] White H , LaFleur J , Houle K , Hyry‐Dermith P , Blake SM . Evaluation of a school‐based transition program designed to facilitate school reentry following a mental health crisis or psychiatric hospitalization. Psychol Sch. 2017;54(8):868‐882. doi:10.1002/pits.22036

[35] Jordan JR . Postvention is prevention‐the case for suicide postvention. Death Stud. 2017;41(10):614‐621. doi:10.1080/07481187.2017.1335544 28557579

[36] O'Neill JC , Marraccini ME , Bledsoe SE , Knotek SE , Tabori AV . Suicide postvention practices in schools: school psychologists' experiences, training, and knowledge. Sch Psychol. 2020;35(1):61‐71. doi:10.1037/spq0000331 31424242

[37] Walling MA . Suicide contagion. Curr Trauma Rep. 2021;7(4):103‐114. doi:10.1007/s40719-021-00219-9 34931156 PMC8674834

[38] Utah State Board of Education . Bullying, Cyber Bullying, Hazing, and Retaliation Model Policy. Utah State Board of Education; 2018. Accessed March 12, 2023. https://www.schools.utah.gov/file/86110147‐0c87‐43be‐a6cd‐21617e053cf5

