# Supplementary material for: The School Suicide Policy Evaluation Tool (SSPET): A proof‐of‐concept for assessing school suicide prevention policies
Source: Public Health Chall. 2024 May 2;3(2):e178. doi: 10.1002/puh2.178 (PMC12039612; doi:10.1002/puh2.178)
Supplement: Supplementary file 1 — Supporting Information [file PUH2-3-e178-s001.pdf]

Table S1: Directory of District Suicide Prevention Policies	
**Alpine**	https://docs.google.com/document/d/1dokLFUt0zvROAAssIsHWpt2w2O_E-zA8C7RPjtZL2YY/edit
**Beaver**	https://www.beaver.k12.ut.us/district/policies/
**Box Elder**	https://core-docs.s3.amazonaws.com/documents/asset/uploaded_file/1383964/5360-Suicide_Prevention.pdf
**Cache County**	https://go.boarddocs.com/ut/ccsdut/Board.nsf/Public?open&id=policies#
**Canyons**	https://www.canyonsdistrict.org/policies/student/
**Carbon**	https://go.boarddocs.com/ut/carbon/Board.nsf/Public#
**Daggett**	https://www.dsdf.org/files/user/5/file/Board%20Policies/Article%20F/FDB%2011-24-20.pdf
**Davis**	https://resources.finalsite.net/images/v1533112542/davisk12utus/xufmuuja84yvsh7lvg4q/5S-100StudentConductandDiscipline.pdf
**Duchesne**	https://drive.google.com/file/d/1TtNV3cIbZGOQAf6FNDHXnXnhYdiqgkvg/view
**Emery County**	https://policies.emeryschools.org/JLDBA-SuicidePrevention
**Garfield County**	https://drive.google.com/drive/folders/1sCG5c55POK0EfZbYbdSlbCQja37YHQI9
**Grand County**	https://docs.google.com/document/d/1Mjap-oQ5DzAVHM0K6OvXJuXHDovkNlKvvnvN9se1gwQ/edit
**Granite**	https://cdn-59bd6cf5f911c923e82ee0ee.closte.com/legal/wp-content/uploads/sites/22/2019/12/VIII.B.9.-Prohibition-of-Bullying-Cyberbullying-Hazing.pdf
**Iron County**	https://sites.google.com/sedck12.org/icsd-policies-and-handbooks/district/section-j/jfbaa-suicide-prevention-policy?authuser=0
**Jordan**	https://policy.jordandistrict.org/sub/
**Juab**	https://www.juabsd.org/district/boe/f-students/3389-policy-fdb-health-requirements-youth-suicide-prevention.html
**Kane County**	https://kanek12.org/wp-content/uploads/2021/09/FDB-Health-Requirements-Suicide-Prevention.pdf
**Logan City**	https://www.loganschools.org/fdb-youth-suicide-prevention
**Millard County**	https://www.millardk12.org/wp-content/uploads/Policy/Article6/6105.pdf
**Morgan County**	https://s3.amazonaws.com/scschoolfiles/2167/fgad__student_bullying_harassment_hazing.pdf
**Murray City**	https://go.boarddocs.com/ut/murrayschools/Board.nsf/files/BTPN8P5EB93B/$file/PS_418_BULLYING_HAZING_REV%2006_09_18.pdf
**Nebo**	https://www.nebo.edu/pubpolicy/J/JDD-GBEA.pdf
**North Sanpete**	https://www.nsanpete.org/districtpolicies/districtpoliciesinstruction/2082-vi-50-suicide-prevention-policy.html
**North Summit**	https://core-docs.s3.amazonaws.com/documents/asset/uploaded_file/1330305/FDDD.pdf<br>https://core-docs.s3.amazonaws.com/documents/asset/uploaded_file/1330319/FGAA.pdf
**Ogden City**	https://drive.google.com/file/d/0B1KCNEn8XWpZTjB0dGVsNmRKN1U/view?resourcekey=0-d9ostAG36TLSChrdMYz36w
**Park City**	https://go.boarddocs.com/ut/pcsd/Board.nsf/goto?open&id=ASE58W0F124A#
**Piute County**	https://www.piutek12.org/en/board-of-education/pcsd-policies/17-2000-3000-personnel/41-2089-suicide-prevention.html<br>https://www.piutek12.org/en/board-of-education/pcsd-policies/18-4000-student-policies/311-4090-bullying-cyberbulling-harassment-and-hazing.html
**Provo City**	https://provo.edu/wp-content/uploads/2020/08/Policy-3320-Prohibition-of-Bullying-Harassment-Hazing-and-Retaliation.pdf
**Rich**	https://richschool-my.sharepoint.com/personal/webmaster_richschool_org/_layouts/15/onedrive.aspx?id=%2Fpersonal%2Fwebmaster%5Frichschool%5Forg%2FDocuments%2Fdistrict%2Fpolicies%2F500%20Student%20Activities%2C%20Rights%2C%20%26%20Responsibilities%2Epdf&parent=%2Fpersonal%2Fwebmaster%5Frichschool%5Forg%2FDocuments%2Fdistrict%2Fpolicies
**Salt Lake City**	https://resources.finalsite.net/images/v1624473392/slcschoolsorg/k0eqoq5gbtnptyzlaedr/g-21-administrative-procedures-english.pdf<br>https://resources.finalsite.net/images/v1615917345/slcschoolsorg/s1ijfj5nruzwdi6rodtw/g-21-policy-english.pdf
**San Juan**	http://old.sjsd.org/district-information/board-policy/#students
**Sevier**	https://www.seviersd.org/index.php/parentstudents/topstudent/404-3950-student-rights-a-responsibilities-new.html<br>https://www.seviersd.org/index.php?view=article&id=750:sevier-school-district-emotional-safety-culture-initiatives&catid=381
**South Sanpete**	https://www.ssanpete.org/policies/j-students/634-jga-bullying-and-hazing.html
**South Summit**	https://core-docs.s3.amazonaws.com/documents/asset/uploaded_file/187011/6037SafeSchoolsSuicidePrevention.pdf
**Tintic**	https://www.tintic.org/board-of-education/policies/f-students/462-fdf-students-health-requirements-and-services-youth-suicide-prevention.html
**Tooele County**	https://go.boarddocs.com/ut/tooelesd/Board.nsf/Public#
**Uintah**	https://www.uintah.net/common/pages/DisplayFile.aspx?itemId=4702493
**Wasatch County**	https://www.wasatch.edu/cms/lib/UT01000315/Centricity/Domain/5/Bullying%20Policy%20Wasatch%207.16.17.pdf
**Washington County**	https://procedure.washk12.org/policy/2000/2110
**Wayne County**	https://www.wayne.k12.ms.us/docs/district/new%20folder/wayne%20county%20school%20district%20suicide%20prevention%20policy%209.26.18%20%20final.pdf?id=17255
**Weber**	https://wsd.net/docman-list/documents/board/policies-and-procedures/157-article-5-student-conduct/file
